# *Koolungar* (Children) *Moorditj* (Strong) Healthy Skin Project Part II: Skin Health in Urban‐Living Australian Aboriginal Children

**DOI:** 10.1111/pde.70016

**Published:** 2025-09-12

**Authors:** Bernadette M. Ricciardo, Heather‐Lynn Kessaris, Noel Nannup, Dale Tilbrook, Jacinta Walton, Carol Michie, Brad Farrant, Ainslie Poore, Ingrid Amgarth‐Duff, Nadia Rind, Richelle Douglas, Jodie Ingrey, Hannah Thomas, S. Prasad Kumarasinghe, Jonathan R. Carapetis, Asha C. Bowen

**Affiliations:** ^1^ University of Western Australia Crawley Western Australia Australia; ^2^ The Kids Research Institute Australia Nedlands Western Australia Australia; ^3^ Perth Children's Hospital Nedlands Western Australia Australia; ^4^ Fiona Stanley Hospital Murdoch Western Australia Australia; ^5^ Derbarl Yerrigan Health Services Aboriginal Corporation East Perth Western Australia Australia; ^6^ South West Aboriginal Medical Service Bunbury Western Australia Australia

**Keywords:** Aboriginal, adolescents, children, co‐design, dermatology, indigenous, skin, urban, young people

## Abstract

**Background:**

Although essential for overall health and wellbeing, little is known about skin health in urban‐living Australian Aboriginal children. This co‐designed, research‐service project aimed to describe skin health and document skin disease frequency in urban‐living Aboriginal children and young people (CYP, i.e., 0–18 years) in Western Australia (WA) and investigate housing associations for skin infections.

**Methods:**

Cross‐sectional studies were conducted at Aboriginal Community Controlled Health Organizations in Bunbury and Perth, WA, over 2 weeks in September–October 2022. Aboriginal CYP were eligible to participate. Questionnaire responses and examination findings were analyzed.

**Results:**

Of the 164 CYP recruited, 149 (91%) were urban‐living Aboriginal CYP. Fifty‐three percent (78/148) of caregivers described a dermatological concern in their child; with high caregiver diagnostic accuracy for impetigo (96%), tinea (92%), and atopic dermatitis (AD) (89%). AD (18%, 26/147), head lice (18%, 27/147), tinea (12%, 18/147) and impetigo (7%, 10/147) were most prevalent. Social housing predicted current head lice (odds ratio [OR] 4.63; 95% confidence interval [CI] 1.72–12.50), current tinea (OR 3.15; 95% CI 1.06–9.36) and ever impetigo (2.39; 95% CI 1.09–5.27). Crowded living conditions predicted ever impetigo (OR 6.28; 95% CI 2.03–19.37); whereas frequent bathing (*p* value 0.032) and regular swimming in a chlorinated pool (OR 0.12; 95% CI 0.02–0.95) were protective.

**Conclusions:**

We report high caregiver diagnostic accuracy for skin conditions. AD is prevalent, with undertreatment, frequent impetiginization, and sleep disturbance indicating barriers to care. Healthcare providers must advocate for improved housing, as the link between skin infections and socioeconomic disadvantage impacts overall health for urban‐living Aboriginal CYP.

## 1 Introduction

Despite living in a high‐income country, remote‐living Australian Aboriginal and/or Torres Strait Islander (hereafter, Aboriginal) children carry the highest global burden of impetigo [1]. This stems from social determinants of health shaped by colonization, dispossession, and systemic racism—not inherent risk. This inequitable burden leads to further health inequities, where Aboriginal people account for > 90% of all Australians with rheumatic heart disease despite being < 4% of the population (Appendix A) [2].

Little data exist on skin health in urban‐living Aboriginal children and young people (CYP, i.e., 0–18 years), despite accounting for nearly 60% of Aboriginal CYP in Western Australia (WA) [3]. This group is 10 times more likely to be hospitalized for skin infections than their non‐Aboriginal peers [4], with recurring skin infections affecting > 7% [5]. Data are lacking on other skin conditions including AD, a known risk factor for infections [6]. A systematic review found AD and bacterial skin infections (BSI) to be more prevalent and severe among urban‐living Indigenous children in high‐income countries globally compared to non‐Indigenous [7].

We aimed to describe skin health, document skin disease frequency, and investigate housing associations for skin infections for urban‐living Aboriginal CYP in WA.

## 2 Methods

We reported following STROBE and CONSIDER guidelines. The methods build on the published 2021 Pilot with the addition of a second site [8].

### 2.1 Study Design

The *Koolungar* (children) *Moorditj* (strong) Healthy Skin (KMHS) Project is set in Bunbury and Perth, WA, Australia, where the traditional custodians are the *Noongar* Aboriginal people. Co‐designed with *Noongar* Elders, we conducted cross‐sectional studies of Aboriginal CYP presenting to two urban Aboriginal Community Controlled Health Organizations (ACCHO).

### 2.2 Study Setting

We partnered with the South West Aboriginal Medical Service (SWAMS) on *Wardandi Noongar* (Bunbury) *Boodjar* (land/place) and Derbarl Yerrigan Health Service (Derbarl) on *Whadjuk Noongar* (Perth) *Boodjar*. Both ACCHOs deliver an Aboriginal Health Practitioner (AHP)‐led model of integrated primary care [9]. Together, they serve ~35% (6579/19,077) of Aboriginal CYP in their locality (SWAMS and Derbarl Business Information Units [BIU], unpublished data) [10].

With guidance from *Wardandi* and *Whadjuk* community advisory groups (CAG), the 2022 Screening Weeks occurred during school holidays at each ACCHO: 5 days at SWAMS and 6 days at Derbarl in September–October 2022. The study period aligned with the Noongar seasons of *Djilba* (August–September, wet) and *Kambarang* (October–November, dry). A team of clinicians and researchers conducted the study following cultural awareness training. Participants received skin health education and dermatologist management for identified skin conditions as per the research‐service model.

### 2.3 Participant Selection and Recruitment

Urban‐living CYP of Aboriginal descent were eligible. Pragmatic convenience sampling was used, recruiting participants presenting for any reason, supplemented by ACCHO‐led promotion inviting CYP to participate, regardless of health issues.

### 2.4 Sample Size Calculation

We aimed to recruit a minimum of 139 participants: based on expected impetigo prevalence of 10% in an unlimited population size, with 95% confidence interval (CI) and 5% precision [5, 8].

### 2.5 Data Collection

Caregiver written informed consent was obtained for seven project components: (1) caregiver questionnaire [11]; (2) height/weight measurements to calculate body mass index (BMI); (3) clinician‐determined Fitzpatrick skin phototype (FSP) and examination of exposed skin/hair/nails; (4) examination of skin concern on covered site(s) facilitated with privacy; (5) clinical photos of skin condition(s); (6) skin cultures for suspected impetigo; and (7) educational presentation and quiz [12].

### 2.6 Definitions

Urban was defined as residence in Modified Monash category one (MM1; metropolitan areas) or two (MM2; regional centers) [13]. Crowded living conditions were defined as persons per bedroom (PPB) > 2 [14]. Other variable definitions are outlined in footnotes of Appendix B: Tables B1, B2, B3, B4.

### 2.7 Data Analysis and Synthesis

Data were entered into REDCap and analyzed using R version 4.1.2. Summary statistics of participant demographics, skin health practices, and medical history were calculated. Lifetime and point prevalence of dermatological diagnoses were calculated, and where seamlessly identical, the data were pooled with the 2021 Pilot [8]. Sensitivity, specificity, and accuracy of caregiver‐reported dermatological concerns and *current eczema symptoms* (International Study of Asthma and Allergies in Childhood, ISAAC) questionnaire were compared with dermatologist assessment [15].

Fisher's exact test investigated housing conditions associated with different housing types, reported with odds ratios (OR) and 95% CI (± *p* values where CI borderline). Prevalence OR and 95% CI investigating disease associations for head lice, tinea, impetigo, and AD by housing type and conditions, skin care routine, perinatal and past medical history, BMI, and skin examination findings were calculated. From these, multivariate logistic regression (generalized linear model) investigated disease associations if ≥ 2 significant predictors.

## 3 Results

**FIGURE 1:**
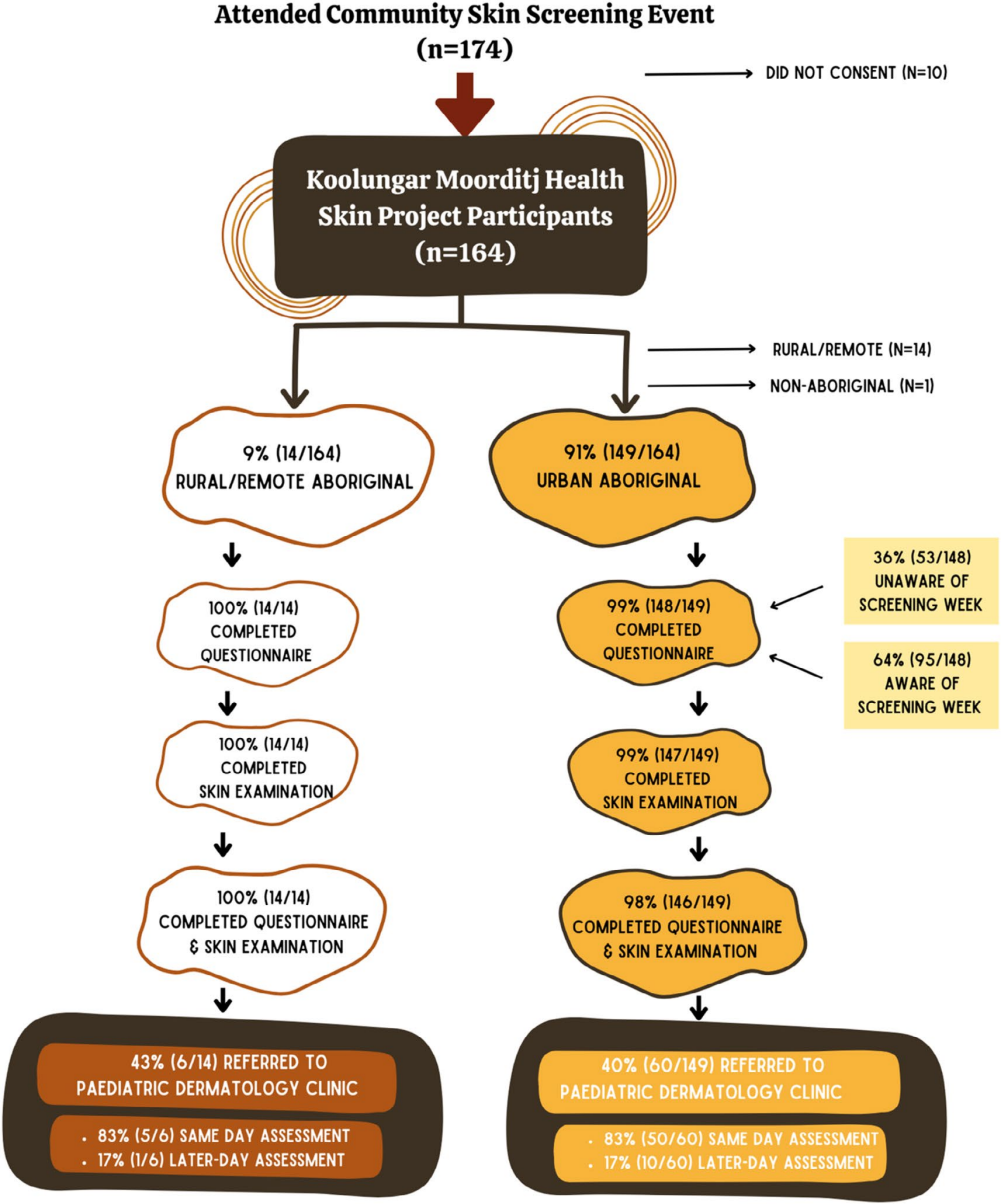
Participant selection and research‐service pathway.

Of 174 potential participants, 164 CYP consented (94%), of whom 149 (91%) were urban‐living Aboriginal CYP and contribute to this analysis (Figure 1): median age 6 years (interquartile range [IQR] 2.75–9 years), 56% (92/164) female (Table 1). Thirty‐six percent (54/149) attended unaware of the screening week, whereas 64% (95/149) participated after promotion. Ninety‐nine percent completed the questionnaire (148/149) and examination (147/149).

**TABLE 1:** Participant demographics.

	Total (*n* = 164)
Indigenous status	
Aboriginal	159 (97%)
Aboriginal and Torres Strait Islander	4 (2%)
Neither Aboriginal or Torres Strait Islander	1 (1%)
Geographical location of usual residence	
Urban^a^	150 (91%)
Rural/remote^b^	14 (9%)
Geographical location of place of birth	
Urban^a^	149 (91%)
Rural/remote^b^	13 (8%)
Unknown	2 (1%)
Sex	
Male	72 (44%)
Female	92 (56%)
Age group	
0 to < 10 years	125 (76%)
10 years to < 19 years	39 (24%)
Median age (IQR)	6 years (2.75, 9)

Abbreviations: CYP, children and young people; MM, modified Monash category.

^a^ Urban‐living defined as MM 1 (metropolitan areas) and MM 2 (regional centers).

^b^ Rural/remote defined as MM3 (large rural towns), MM4 (medium rural towns), MM5 (small rural towns), MM6 (remote communities) and MM7 (very remote communities).

### 3.1 Skin Health Measures

#### 3.1.1 Skin Care Routine

Frequent bathing with bathing agents was reported in 99% (146/148); soap, shower‐gel, or shampoo (107/146, 73%) more commonly than soap‐free wash (39/146, 27%). Forty‐seven percent (70/148) applied emollient frequently. Eleven percent (17/148) regularly swam in a chlorinated pool, and 6% (9/148) in the ocean.

#### 3.1.2 Aboriginal Bush Medicine

Ten percent (15/148) used topical bush medicine for daily skin care; 53% (8/15) described remedies with traditional oils (e.g., emu, kangaroo, sandalwood) or contemporary ingredients (e.g., aloe vera, honey), whereas 47% (7/15) chose not to discuss active ingredients.

Forty‐one percent (61/148) used bush medicine for skin problems: 49% (30/61) topical, 5% (3/61) oral/topical, and 46% (28/61) chose not to comment. Fifty‐four percent (33/61) described remedies with traditional oils (e.g., emu, kangaroo, sandalwood, eucalyptus, tea tree) and contemporary ingredients (e.g., aloe vera, calendula oil). Twenty‐three percent (14/61) were unsure of ingredients but knew who to consult for the specific concern: mostly “sores,” “irritation,” “itch,” or “sunburn.”

#### 3.1.3 Sun‐Protection

**FIGURE 2:**
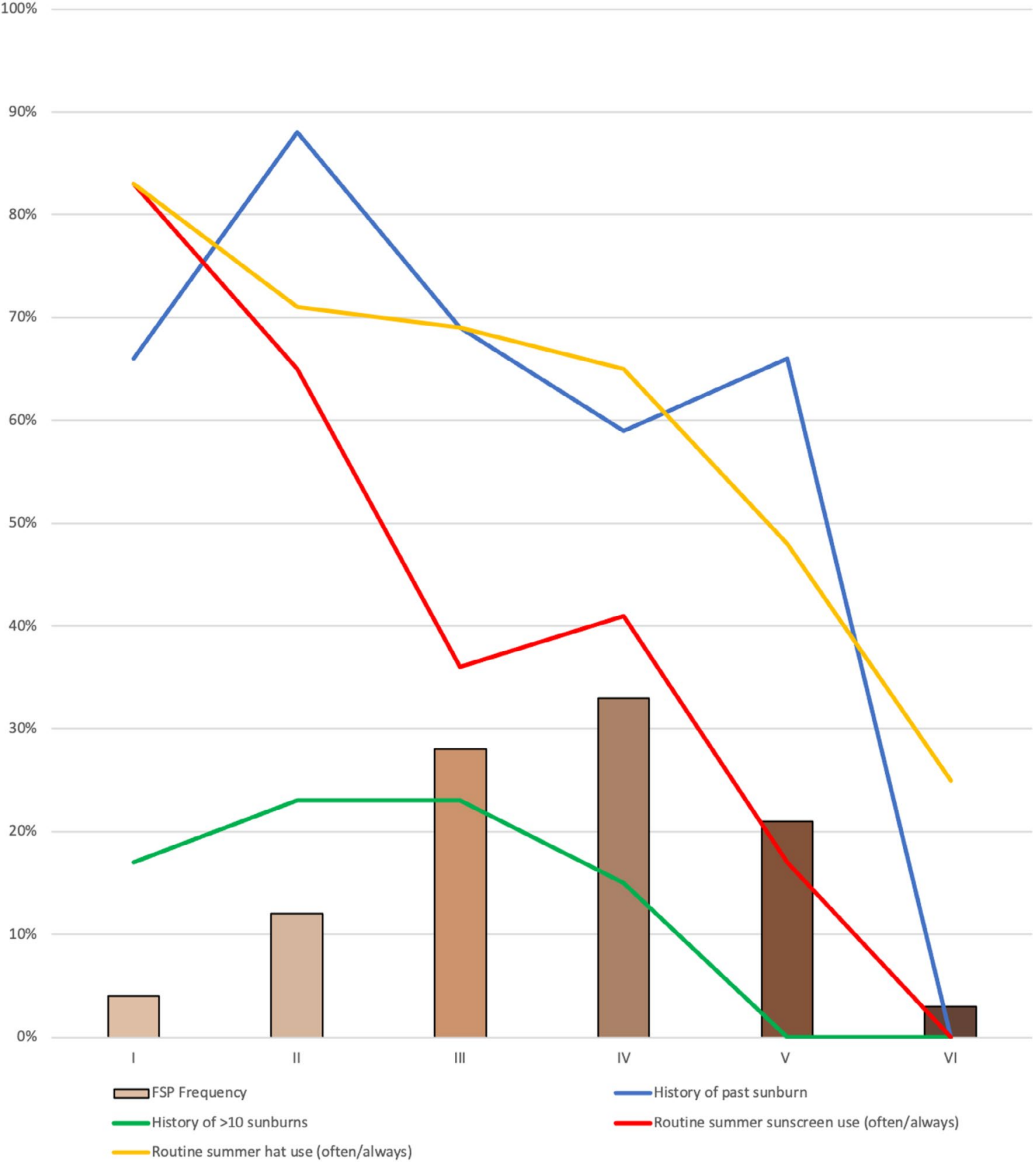
Fitzpatrick skin phototype (FSP), sunburn and sun safety behaviors among urban‐living Aboriginal CYP.

FSP was dermatologist‐determined for 95% (141/149) (Figure 2). Previous sunburn was present in 65% (92/141); highest in FSP II (15/17, 88%) and III (27/39, 69%), where 23% (13/56) reported > 10 sunburns. Sixty‐three percent (89/141) wore hats routinely in summer. Thirty‐eight percent (54/141) used sunscreen routinely in summer; reasons for sporadic use (84/141, 60%) included: “not part of routine” (60/102, 59%), “intolerance” (15/102, 15%), “skin doesn't burn” (6/102, 6%), “other sun‐protection preferred” (6/102, 6%), and cost (5/102, 5%).

#### 3.1.4 Housing

Most CYP resided in social housing (48/148, 32%), rental property (42/148, 28%), own home (32/148, 22%), temporary accommodation (13/148, 9%), transitional accommodation (5/148, 3%), or unsure/no answer (8/148, 5%). Residents per household ranged from 2 to 11 (median: 5; IQR: 4–6). Eleven percent (16/147) experienced crowded living conditions; 8‐fold (OR 8.17; 95% CI 1.58–81.15) more likely in social housing compared with own home/rental. Sixty‐two percent (92/148) described bed‐sharing: 20% (18/92) by necessity and 78% (72/92) by preference (unsure, *n* = 2).

Three percent (5/148) lacked a working washing machine; all in social housing, transitional, or temporary accommodation (*p* value 0.021). Ten percent (15/148) had bathroom plumbing/maintenance issues; 3.5‐fold (OR 3.54; 95% CI 1.01–14.21) more likely in social housing compared with own home/rental.

Forty‐five percent (67/148) lived with smokers; 2‐fold (OR 2.17; 95% CI 0.97–4.89; *p* value 0.042) more likely in social housing compared with own home/rental. Sixty percent (89/148) had furry pets at home; 2‐fold (OR 2.53; 95% CI 1.08–6.22) more likely in social housing compared with own home/rental.

### 3.2 Skin Disease Measures

#### 3.2.1 Current Dermatological Concerns

Caregivers reported current dermatological concerns in 53% (78/148): 42% (22/53) in those unaware of the screening week and 59% (56/95) in those who participated following promotion; with dermatological concerns 2‐fold (OR 2.01; 95% CI 0.97–4.24) more likely in the latter. Most reported AD (23/148, 16%), tinea (16/148, 11%), and impetigo (7/148, 5%); with 89%, 92%, and 96% caregiver diagnostic accuracy, respectively.

#### 3.2.2 Skin Disease Prevalence

Lifetime (Table 2) and point prevalence (Table 3) of dermatological diagnoses are presented.

**TABLE 2:** Lifetime prevalence of dermatological (and associated) diagnoses in urban‐living^a^ Aboriginal CYP from questionnaire.

	KMHS 2021 [8], *n* (%)	KMHS 2022, *n* (%)	KMHS 2021 + 2022, *n* (%)
Total number of participants	80	148	228
“Has your child ever had…”			
Infections			
Impetigo	34 (43%)	45 (30%)	79 (35%)
Tinea^b^	30 (38%)	46 (31%)	76 (33%)
Scabies	11 (14%)	7 (5%)	18 (8%)
Hospitalization due to impetigo complication			
Cellulitis or abscess	0	5 (3%)	5 (2%)
Bone or joint infection	2 (3%)	0	2 (1%)
Acute rheumatic fever	0	1 (1%)	1 (0.5%)
“Has your child every been diagnosed by a doctor with…”			
Atopy			
Atopic dermatitis	15 (19%)	19 (13%)	34 (15%)
Asthma	13 (16%)	17 (11%)	30 (13%)
Hay fever	9 (11%)	14 (9%)	23 (10%)
Food allergy	1 (1.2%)	8 (5%)	9 (4%)
Deficiency			
Iron deficiency	13 (16%)	20 (14%)	33 (14%)
Vitamin D deficiency	3 (4%)	16 (11%)	19 (8%)

Abbreviations: CYP, children and young people; KMHS, Koolungar Moorditj Healthy Skin Project.

^a^ Urban‐living defined as MM 1 (metropolitan areas) and MM 2 (regional centers).

^b^ Tinea includes tinea corporis, tinea capitis, and onychomycosis.

**TABLE 3:** Point prevalence of dermatological diagnoses in urban‐living^a^ Aboriginal CYP on examination.

	KMHS 2021 [8], *n* (%)	KMHS 2022, *n* (%)	KMHS 2021 + 2022, *n* (%)
Total number of participants	79	147	226
Infectious dermatological disorders			
Head lice	18 (23%)	27 (18%)	45 (20%)
Tinea (total)	15 (19%)	18 (12%)	33 (15%)
Tinea corporis	9 (11%)	15 (10%)	24 (11%)
Tinea capitis	8 (10%)^b^	8 (5%)^c^	16 (7%)
Onychomycosis	2 (2%)	0	2 (1%)
Viral verrucae	4 (5%)	14 (10%)	18 (8%)
Impetigo (total)	4 (5%)^d^	10 (7%)^e^	14 (6%)
Primary infection	2 (2%)	5 (3%)	7 (3%)
Secondary infection of AD	2 (2%)	5 (3%)	7 (3%)
Scabies—classic	1 (1%)	4 (3%)	5 (2%)
Molluscum contagiosum	0	4 (3%)	4 (2%)
Non‐infectious dermatological disorders			
Atopic dermatitis (eczema)	12 (15%)	26 (18%)	38 (17%)
Secondary BSI of atopic dermatitis	2/12 (17%)	5/26 (19%)	7/38 (18%)
Keratosis pilaris	8 (10%)	24 (16%)	32 (14%)
Seborrhoeic dermatitis	11 (14%)	21 (14%)	32 (14%)
Acne	18 (23%)	9 (6%)	27 (12%)
0 to 9‐year olds	0	1/111 (1%)	1/157 (1%)
10 to 19‐year olds	18/33 (55%)	8/36 (22%)	26/69 (38%)
Pityriasis alba	10 (13%)	14 (10%)	24 (11%)
Post‐inflammatory dyspigmentation^f^	—	13 (9%)	—
Hypopigmentation	—	8 (5%)	—
Hyperpigmentation	—	5 (3%)	—
Arthropod bites (mosquitos, fleas)	0	12 (8%)	0
Hypertrophic and/or keloid scarring	0	10 (7%)	0
“Other” dermatitis^g^	6 (8%)	10 (7%)	16 (7%)
Acral naevi	5 (6%)	8 (5%)	13 (6%)
Nail disorders (non‐infectious)	0	8 (5%)	0
Acanthosis nigricans ± acrochordons	1 (1%)	8 (5%)	9 (4%)
Ichthyosis (including IV, XLRI)	1 (1%)	4 (3%)	5 (2%)

Abbreviations: AD, atopic dermatitis; BSI, bacterial skin infection; CYP, children and young people; KMHS, Koolungar Moorditj Healthy Skin Project.

^a^ Urban‐living defined as MM 1 (metropolitan areas) and MM 2 (regional centers).

^b^ *Trichophyton tonsurans* cultured from 100% (6/6) of six hair plucks collected.

^c^ *T. tonsurans* cultured from 75% (6/8) of eight hair plucks collected.

^d^ *Staphylococcus aureus* (*n* = 4) cultured from four skin swabs collected.

^e^ *S. aureus* (*n* = 7) and *Streptococcus pyogenes* (*n* = 1) cultured from 10 skin swabs collected.

^f^ Post‐inflammatory dyspigmentation was not recorded in the 2021 Pilot.

^g^ “Other” dermatitis—includes phytophotodermatitis, lip‐licker dermatitis, diaper dermatitis, frictional lichenoid dermatitis, lichen simplex chronicus, molluscum dermatitis.

#### 3.2.3 Atopic Dermatitis

*ISAAC Current eczema symptoms* were present in 15% (22/148); demonstrating 75% diagnostic accuracy compared with dermatologist diagnosis on examination (sensitivity 93%, specificity 62%: false positives in scabies [*n* = 3], head lice [*n* = 2], “other” dermatitis [*n* = 3]). Of those with both *current eczema symptoms* and AD on examination (true positives, *n* = 14), 43% (6/14) reported *severe eczema symptoms*. Of CYP with AD on examination, 42% (11/26) were using soap‐free wash, 31% (8/26) daily emollient, and 27% (7/26) topical corticosteroid.

### 3.3 Housing, Swimming and Bathing Associations of Skin Infections (Appendix B)

#### 3.3.1 Head Lice

Living in social housing compared with own home/rental (OR 4.63; 95% CI 1.72–12.50) predicted *current* head lice.

#### 3.3.2 Impetigo

Living in social housing compared with own home/rental (OR 2.39; 95% CI 1.09–5.27) and crowded living conditions (OR 6.28; 95% CI 2.03–19.37) predicted *ever* impetigo. Multivariate models confirmed the harm of crowded living conditions (OR 5.86; 95% CI 1.5–29.05). Swimming regularly in a chlorinated pool (OR 0.12; 95% CI 0.02–0.95) reduced impetigo risk, and 100% of CYP without *ever* impetigo bathed frequently (*p* value 0.032). Multivariate models confirmed the benefit of swimming regularly (OR 0.13; 95% CI 0.01–0.66). Frequent bathing (*p* value 1.66e‐07) was associated with a lower risk of impetigo, whereas bedsharing (*p* value 0.012) was associated with impetigo risk; with significance lost when analyzed for bedsharing by necessity (*p* value 0.396).

#### 3.3.3 Tinea

Living in homes with plumbing/bathroom maintenance issues (OR 2.90; 95% CI 0.98–8.58, *p* value 0.047) predicted *ever* tinea. Living in social housing compared with own home/rental (OR 3.15; 95% CI 1.06–9.36) and furry pets at home (OR 3.75; 95% CI 1.03–13.61) predicted *current* tinea. Multivariate models did not confirm the statistical significance of these risk factors.

## 4 Discussion

In this community‐based study of urban‐living Aboriginal CYP:

1. Over half of caregivers reported a current dermatological concern for their child; accurately identifying impetigo, tinea, and AD.
2. AD and skin infections were most prevalent, with housing linked to skin infections.
3. Sunburn was common, especially in CYP with lighter phototypes.
4. Traditional and contemporary bush medicines were widely used for skin health.

Current dermatological concerns were common, aligning with the 2021 Pilot [8]. Caregivers most often cited AD, tinea, and impetigo, with high diagnostic accuracy. Caregiver recognition of AD (89%) exceeded that of the ISAAC questionnaire (75%), highlighting strong caregiver knowledge despite risks of misclassification with other pruritic conditions. Although utilized in other First Nations pediatric populations, further validation of the ISAAC questionnaire is needed for Aboriginal families, considering cultural and literacy contexts [7, 15].

AD was the most prevalent non‐infectious skin condition, affecting 18% of CYP. One in five cases was complicated by secondary BSI. Although prevalence aligns with national rates for CYP, AD undertreatment was common, with only one‐third using daily emollients and topical corticosteroids [16]. Almost half with AD experienced *severe eczema symptoms*, causing sleep disturbance. To improve care, our team published the *National Healthy Skin Guideline* (NHSG) (thekids.org.au) second edition with an AD chapter, co‐designed a caregiver AD factsheet, and created a children's storybook, *Kaal Tackles Eczema* [12, 16, 17].

Housing conditions, previously linked to skin infections in remote settings, were also relevant in urban areas [18]. Compared to Census data, study families had lower rates of home ownership and higher rates of social housing, where lack of working washing machines, plumbing/maintenance issues, and crowded conditions increased risks for head lice, tinea, and impetigo [19]. Crowded living, reported in 11% of households, predicted impetigo risk [19]. These findings reinforce housing as a social determinant of skin health and support advocacy for improved social housing and maintenance—an Elder Researcher priority [20, 21].

Regular swimming and bathing were protective against impetigo. Chlorinated pool use aligns with previous evidence from remote Aboriginal communities, whereas daily bathing echoes global findings linking hygiene to reduced infections [22, 23]. These messages have been incorporated into culturally appropriate health promotion resources co‐designed with Elder Researchers and CAGs (https://www.thekids.org.au/our‐research/infectious‐diseases/healthy‐skin‐and‐arf‐prevention/resource‐hub/) [12].

Sunburn was common, particularly among CYP with lighter phototypes (FSP II/III). Of this group, 75% had experienced sunburn, and nearly one‐quarter reported > 10 lifetime sunburns. Sunscreen and hat use were suboptimal. The only study on skin cancer in Aboriginal populations found urban‐living adults with FSP II/III made up 86% of Aboriginal skin cancer cases, often with delayed diagnosis [24]. Improved sun safety education is needed, tailored to Aboriginal CYP of all skin tones.

Bush medicine, part of Aboriginal health practices for > 65,000 years, remains relevant for skin health, with 10% of CYP using it daily [25]. Published records reveal numerous ethnomedicines traditionally used for skin conditions, with studies revealing their pharmacological properties [26]. In our study, 40% of CYP used bush medicine for skin problems; primarily topical preparations with plant extracts or animal oils. Although ingredients were often withheld—likely due to cultural protocols or concerns of exploitation—these traditional knowledges present therapeutic opportunities if guided and governed by Aboriginal communities [27].

### 4.1 Limitations

A strength of this study was the research‐service model as it addressed health inequities in real time [9]. However, recruitment within healthcare facilities may have introduced selection bias and impacted generalizability; as ACCHOs may not represent all urban‐living Aboriginal CYP. Despite promotion beyond ACCHOs and a focus on healthy skin, self‐selection bias likely skewed results toward skin disease. Other potential limitations include recall and seasonal biases in reporting, and limited power to assess disease associations due to small sample sizes.

## 5 Conclusion

This community‐based description of skin health in urban‐living Aboriginal CYP highlights high caregiver diagnostic accuracy and frequent bush medicine use for prevention and treatment. AD is prevalent, with undertreatment, secondary BSI, and sleep disturbance indicating barriers to care. Healthcare providers must advocate for improved housing, as the link between skin infections and socioeconomic disadvantage impacts overall health for urban‐living Aboriginal CYP. Partnering with Aboriginal Elders, families, and communities is essential to addressing inequities and improving skin health.

## Author Contributions

Each named author has substantially contributed to the creation of this manuscript as per the International Committee of Medical Journal Editors recommendations for authorship.

## Ethics Statement

Ethics approval for this study was provided by the WA Aboriginal Health Ethics Committee (HREC ref. no. 1059) and the University of WA (file reference—2021/ET000536). All parents gave written informed consent. All participants gave verbal assent prior to skin examination.

## Conflicts of Interest

The authors declare no conflicts of interest.

## Data Availability

The data that support the findings of this study are available on request from the corresponding author. The data are not publicly available due to privacy or ethical restrictions.

## Appendix B Disease Associations for Common Childhood Dermatological Diagnoses

**TABLE B1:** Head lice: disease associations in urban‐living Aboriginal CYP.

Response category	Current head lice (*n* = 27)		
	*n* (%)	OR (95% CI)	*p*
Household structure			
Crowded living conditions^a^	3 (11%)	1.01 (0.27, 3.82)	0.989
Stable household numbers	26 (96%)	3.75 (0.47, 29.70)	0.183
Bedsharing: sometimes or always^b^	19 (70%)	1.52 (0.61, 3.75)	0.366
Bedsharing by necessity^b^	5 (19%)	1.72 (0.52, 5.66)	0.374
Household smokers	15 (56%)	1.61 (0.69, 3.73)	0.266
Household furry pets^c^	18 (67%)	1.60 (0.64, 3.97)	0.312
Social housing, transitional or temporary accommodation^d^	**18 (67%)**	**3.75 (1.45, 9.69)**	**0.005**
Social housing^d^	**15 (56%)**	**4.63 (1.72, 12.50)**	**0.002**
Household health hardware			
Absence of a working washing machine at home	0	0	0.280
Plumbing or bathroom maintenance issues at home	2 (7%)	0.64 (0.14, 3.02)	0.572
Skin care routine			
Frequent bathing^e^	27 (100%)	0	0.499
Use of bathing agent^f^	27 (100%)	Infinite	0.499
Frequent emollient application^g^	12 (44%)	0.8 (0.34, 1.86)	0.604
Regularly swim in a chlorinated pool^h^	1 (4%)	0.25 (0.03, 1.93)	0.152
Regularly swim in the ocean^h^	0	0	0.142
Use of traditional medicinal plants as part of everyday skin care routine	3 (11%)	1.13 (0.30, 4.33)	0.859
Perinatal history			
Maternal age < 20 years at birth	4 (15%)	0.71 (0.22, 2.26)	0.563
Maternal smoking in pregnancy	16 (59%)	2.05 (0.84, 5.01)	0.114
Maternal multiparity^i^	8 (30%)	0.68 (0.28, 1.69)	0.409
Low birth weight^j^	7 (26%)	1.99 (0.73, 5.47)	0.176
Very remote location of birth^k^	0	0	0.332
Past medical history			
Bacterial skin or soft tissue infection (all)	6 (22%)	0.61 (0.23, 1.63)	0.323
Tinea (all)	8 (30%)	0.93 (0.37, 2.32)	0.882
Scabies	0	0	0.450
Eczema (atopic dermatitis)	3 (11%)	0.82 (0.22, 3.06)	0.772
Asthma	2 (7%)	0.56 (0.12, 2.59)	0.451
Hayfever	0	0	0.057
Food allergy	1 (4%)	0.63 (0.07, 5.39)	0.675
Iron deficiency	4 (15%)	1.19 (0.36, 3.92)	0.775
Vitamin D deficiency	3 (11%)	1.07 (0.28, 4.07)	0.921
Body mass index			
Underweight^l^	2 (7%)	0.41 (0.09, 1.87)	0.237
At risk of overweight/overweight/obese^l^	**18 (67%)**	**2.41 (1.00, 5.79)**	**0.046**
Overweight/obese^l^	**16 (59%)**	**2.57 (1.09, 6.04)**	**0.028**
Obese^l^	**12 (44%)**	**3.94 (1.59, 9.80)**	**0.002**
Skin examination findings			
Impetigo (all)	3 (11%)	2 (0.48, 8.29)	0.333
Tinea (all)	6 (22%)	2.55 (0.86, 7.55)	0.084
Scabies	1 (4%)	1.48 (0.15, 14.87)	0.735
AD	3 (11%)	0.52 (0.14, 1.88)	0.315

*Note*: The bold values are the statistically significant results.

Abbreviations: AD, atopic dermatitis; OR (95% CI), prevalence odds ratios with 95% confidence intervals.

^a^ *Crowded living conditions* defined as > 2.0 persons‐per‐bedroom (PPB).

^b^ *Bedsharing* defined as sometimes or always bedsharing, as compared with never. Bedsharing “by necessity” includes reasons such as poor heating or insufficient beds.

^c^ *Household furry pets* include cats, dogs, rabbits, guinea pigs, and mice.

^d^ *Social housing* refers to government subsidized short and long‐term rental housing; mainly available to people on very low incomes, and who often have experienced homelessness, family violence or have other complex needs. Social housing is made up of two types of housing: (a) public housing, which is owned and managed by State and Territory Governments, and (b) community housing, which is managed (and often owned) by not‐for‐profit organizations. *Transitional accommodation* is a form of social housing offered by community housing providers. Funded by the State Governments. It offers tenancies to people on the housing register with a “very high” or “high and urgent need for housing.” *Temporary accommodation* is a broad term that describes temporary housing for people who are homeless. This includes hostels (i.e., Salvation army), bed and breakfasts, hotels, boarding (with family, friends or other), couch surfing, and so forth [Australian Housing and Urban Research Institute].

^e^ *Frequent bathing* is defined as bathing at least once daily.

^f^ *Use of bathing agent* defined as any bathing agent including soap, shower gel, shampoo, bush medicine soap, soap‐free wash or soap substitute (as compared with just water).

^g^ *Frequent emollient application* defined as at least weekly.

^h^ *Regularly swim* defined as at least monthly.

^i^ *Maternal multi‐parity* defined as ≥ 3 previous pregnancies.

^j^ *Low birth weight* defined as < 2500 g or < 5.5 pounds.

^k^ *Very remote location of birth* defined as remote (MM 6) and very remote communities (MM 7).

^l^ Body mass index (BMI)‐for‐age *Z*‐scores, as per the WHO Child Growth Standards, are used to assess whether a child's growth measurements are in a normal range. Cut‐off points of ≤ 1.0, > 1.0, > 2.0, and > 3.0 are used to indicate underweight, at risk of underweight, overweight, and obese, respectively, in children aged 0–5 years. Cut‐off points of ≤ 1.0, > 1.0, and > 2.0 are used to indicate underweight, overweight, and obese, respectively, in CYP aged 5–19 years.

**TABLE B2:** Impetigo: disease associations in urban‐living Aboriginal CYP.

Response category	Ever (past history of) impetigo (*n* = 45)			Current impetigo (*n* = 10)		
	*n* (%)	OR (95% CI)	*p*	*n* (%)	OR (95% CI)	*p*
Household structure						
Crowded living conditions^a^	**11 (24%)**	**6.28 (2.03, 19.37)**	**0.0004**	1 (10%)	0.89 (0.11, 7.51)	0.914
Stable household numbers	37 (82%)	0.39 (0.14, 1.11)	0.073	9 (90%)	1.12 (0.13, 9.43)	0.920
Bedsharing: sometimes or always^b^	31 (69%)	1.49 (0.71, 3.13)	0.296	**10 (100%)**	**Infinite**	**0.012**
Bedsharing by necessity^b^	5 (11%)	0.72 (0.23, 2.26)	0.578	1 (10%)	0.41 (0.05, 3.43)	0.396
Household smokers	17 (38%)	0.64 (0.31, 1.32)	0.228	3 (30%)	0.48 (0.12, 1.94)	0.298
Household furry pets^c^	31 (69%)	1.81 (0.85, 3.86)	0.124	9 (90%)	6.46 (0.80, 52.47)	0.048
Social housing, transitional or temporary accommodation^d^	**26 (58%)**	**2.18 (1.05, 4.53)**	**0.036**	6 (60%)	2.45 (0.59, 10.22)	0.208
Social housing^d^	**20 (44%)**	**2.39 (1.09, 5.27)**	**0.029**	3 (30%)	1.65 (0.32, 8.55)	0.548
Household health hardware						
Absence of a working washing machine at home	0	0	0.134	0	0	0.539
Plumbing or bathroom maintenance issues at home	7 (16%)	2.31 (0.78, 6.83)	0.124	1 (10%)	1.08 (0.13, 9.29)	0.944
Skin care routine						
Frequent bathing^e^	**43 (96%)**	**Infinite**	**0.032**	**8 (80%)**	**Infinite**	**1.664e‐07**
Use of bathing agent^f^	44 (98%)	0.43 (0.03, 7.05)	0.546	10 (100%)	Infinite	0.700
Frequent emollient application^g^	19 (42%)	0.70 (0.35, 1.43)	0.330	7 (70%)	2.59 (0.64, 10.46)	0.169
Regularly swim in a chlorinated pool^h^	**1 (2%)**	**0.12 (0.02, 0.95)**	**0.019**	1 (10%)	0.83 (0.10, 6.96)	0.861
Regularly swim in the ocean^h^	5 (11%)	3.09 (0.79, 12.12)	0.092	0	0	0.403
Use of traditional medicinal plants as part of everyday skin care routine	7 (16%)	2.10 (0.71, 6.19)	0.175	0	0	0.261
Perinatal history						
Maternal age < 20 years at birth	6 (13%)	0.61 (0.23, 1.63)	0.323	2 (20%)	1.09 (0.22, 5.45)	0.917
Maternal smoking in pregnancy	23 (51%)	1.21 (0.60, 2.48)	0.595	6 (60%)	1.57 (0.42, 5.83)	0.498
Maternal multiparity^i^	13 (29%)	0.58 (0.27, 1.24)	0.157	3 (30%)	0.73 (0.18, 2.95)	0.657
Low birth weight^j^	9 (20%)	1.29 (0.52, 3.20)	0.582	4 (40%)	3.12 (0.81, 12.00)	0.085
Very remote location of birth^k^	2 (4%)	2.30 (0.31, 16.88)	0.401	0	0	0.581
Past medical history						
Bacterial skin or soft tissue infection (all)	—	—	—	5 (50%)	2.49 (0.68, 9.07)	0.158
Tinea (all)	17 (38%)	1.55 (0.74, 3.25)	0.246	3 (30%)	0.96 (0.24, 3.89)	0.954
Scabies	3 (7%)	1.74 (0.37, 8.15)	0.477	0	0	0.450
Eczema (atopic dermatitis)	9 (20%)	2.25 (0.84, 5.99)	0.100	3 (30%)	3.13 (0.74, 13.36)	0.108
Asthma	5 (11%)	0.93 (0.31, 2.82)	0.898	0	0	0.2530
Hayfever	5 (11%)	1.35 (0.42, 4.28)	0.616	0	0	0.280
Food allergy	1 (2%)	0.33 (0.04, 2.74)	0.281	1 (10%)	2.02 (0.22, 18.22)	0.526
Iron deficiency	9 (20%)	2.06 (0.78, 5.39)	0.139	3 (30%)	2.87 (0.68, 12.19)	0.138
Vitamin D deficiency	6 (13%)	1.44 (0.49, 4.26)	0.506	1 (10%)	0.85 (0.10, 7.20)	0.883
Body mass index	*n* = 44					
Underweight^l^	6 (14%)	0.83 (0.30, 2.31)	0.724	3 (30%)	2.62 (0.62, 11.07)	0.178
At risk of overweight/overweight/obese^l^	21 (48%)	0.91 (0.45, 1.85)	0.802	3 (30%)	0.42 (0.10, 1.68)	0.207
Overweight/obese^l^	19 (43%)	1.18 (0.58, 2.41)	0.655	3 (30%)	0.61 (0.15, 2.47)	0.488
Obese^l^	11 (25%)	1.16 (0.50, 2.68)	0.731	2 (20%)	0.82 (0.16, 4.06)	0.805
Skin examination findings	*n* = 44					
Head lice	6 (14%)	0.61 (0.23, 1.63)	0.323	3 (30%)	2 (0.48, 8.29)	0.333
Tinea (all)	8 (18%)	2.04 (0.75, 5.59)	0.159	3 (30%)	3.46 (0.81, 14.81)	0.079
Scabies	2 (5%)	2.38 (0.32, 17.47)	0.382	1 (10%)	4.93 (0.46, 52.26)	0.146
AD	10 (23%)	1.58 (0.65, 3.83)	0.309	2 (20%)	1.17 (0.23, 5.84)	0.852

*Note*: The bold values are the statistically significant results.

^a^ *Crowded living conditions* defined as > 2.0 persons‐per‐bedroom (PPB).

^b^ *Bedsharing* defined as sometimes or always bedsharing, as compared with never. Bedsharing “by necessity” includes reasons such as poor heating or insufficient beds.

^c^ *Household furry pets* include cats, dogs, rabbits, guinea pigs, and mice.

^d^ *Social housing* refers to government subsidized short and long‐term rental housing; mainly available to people on very low incomes, and who often have experienced homelessness, family violence or have other complex needs. Social housing is made up of two types of housing: (a) public housing, which is owned and managed by State and Territory Governments, and (b) community housing, which is managed (and often owned) by not‐for‐profit organizations. *Transitional accommodation* is a form of social housing offered by community housing providers. Funded by the State Governments. It offers tenancies to people on the housing register with a “very high” or “high and urgent need for housing.” *Temporary accommodation* is a broad term that describes temporary housing for people who are homeless. This includes hostels (i.e., Salvation army), bed and breakfasts, hotels, boarding (with family, friends or other), couch surfing, and so forth [Australian Housing and Urban Research Institute].

^e^ *Frequent bathing* is defined as bathing at least once daily.

^f^ *Use of bathing agent* defined as any bathing agent including soap, shower gel, shampoo, bush medicine soap, soap‐free wash or soap substitute (as compared with just water).

^g^ *Frequent emollient application* defined as at least weekly.

^h^ *Regularly swim* defined as at least monthly.

^i^ *Maternal multi‐parity* defined as ≥ 3 previous pregnancies.

^j^ *Low birth weight* defined as < 2500 g or < 5.5 pounds.

^k^ *Very remote location of birth* defined as remote (MM 6) and very remote communities (MM 7).

^l^ Body mass index (BMI)‐for‐age *Z*‐scores, as per the WHO Child Growth Standards, are used to assess whether a child's growth measurements are in a normal range. Cut‐off points of ≤ 1.0, > 1.0, > 2.0, and > 3.0 are used to indicate underweight, at risk of underweight, overweight, and obese, respectively, in children aged 0–5 years. Cut‐off points of ≤ 1.0, > 1.0, and > 2.0 are used to indicate underweight, overweight, and obese, respectively, in CYP aged 5–19 years.

**TABLE B3:** Tinea: disease associations in urban‐living Aboriginal CYP.

Response category	Ever (past history of) tinea (*n* = 46)			Current tinea (*n* = 18)		
	*n* (%)	OR (95% CI)	*p*	*n* (%)	OR (95% CI)	*p*
Household structure						
Crowded living conditions^a^	4 (9%)	0.73 (0.22, 2.40)	0.607	2 (11%)	1.00 (0.21, 4.86)	0.991
Stable household numbers	41 (89%)	0.99 (0.32, 3.04)	0.988	14 (78%)	0.36 (0.10, 1.28)	0.104
Bedsharing: sometimes or always^b^	24 (52%)	0.53 (0.26, 1.08)	0.079	14 (78%)	2.27 (0.71, 7.30)	0.1605
Bedsharing by necessity^b^	4 (9%)	0.74 (0.22, 2.53)	0.635	2 (11%)	0.61 (0.12, 3.03)	0.549
Household smokers	20 (43%)	0.90 (0.45, 1.81)	0.769	9 (50%)	1.21 (0.45, 3.24)	0.710
Household furry pets^c^	30 (65%)	1.30 (0.63, 2.69)	0.476	**15 (83%)**	**3.75 (1.03, 13.61)**	**0.034**
Social housing, transitional or temporary accommodation^d^	21 (46%)	1.03 (0.51, 2.11)	0.926	11 (61%)	2.35 (0.82, 6.77)	0.107
Social housing^d^	17 (37%)	1.22 (0.56, 2.63)	0.620	**10 (56%)**	**3.15 (1.06, 9.36)**	**0.033**
Household health hardware						
Absence of a working washing machine at home	0	0	0.128	0	0	0.395
Plumbing or bathroom maintenance issues at home	**8 (17%)**	**2.90 (0.98, 8.58)**	**0.047**	4 (22%)	2.99 (0.84, 10.66)	0.0807
Skin care routine						
Frequent bathing^e^	45 (98%)	0.45 (0.03, 7.28)	0.562	17 (94%)	0.13 (0.01, 2.23)	0.104
Use of bathing agent^f^	46 (100%)	Infinite	0.341	18 (100%)	0	0.595
Frequent emollient application^g^	22 (48%)	0.97 (0.48, 1.96)	0.941	11 (61%)	1.76 (0.64, 4.83)	0.271
Regularly swim in a chlorinated pool^h^	6 (13%)	1.27 (0.44, 3.68)	0.657	3 (0.17)	1.74 (0.44, 6.83)	0.421
Regularly swim in the ocean^h^	4 (9%)	1.85 (0.47, 7.22)	0.373	1 (6%)	0.88 (0.10, 7.50)	0.909
Use of traditional medicinal plants as part of everyday skin care routine	6 (13%)	1.48 (0.49, 4.45)	0.481	2 (11%)	1.07 (0.22, 5.17)	0.936
Perinatal history						
Maternal age < 20 years at birth	11 (24%)	1.72 (0.72, 4.08)	0.218	5 (28%)	1.99 (0.64, 6.22)	0.232
Maternal smoking in pregnancy	22 (48%)	1.06 (0.52, 2.17)	0.865	9 (50%)	1.02 (0.38, 2.74)	0.974
Maternal multiparity^i^	14 (30%)	0.64 (0.30, 1.35)	0.240	8 (44%)	1.46 (0.54, 3.96)	0.459
Low birth weight^j^	**13 (28%)**	**2.79 (1.16, 6.72)**	**0.019**	**7 (39%)**	**4.09 (1.36, 12.33)**	**0.008**
Very remote location of birth^k^	2 (4%)	2.23 (0.30, 16.33)	0.421	0	0	0.445
Past medical history						
Bacterial skin and soft tissue infection (all)	17 (37%)	1.55 (0.74, 3.25)		8 (44%)	2.04 (0.75, 5.59)	0.159
Tinea (all)	—	—		**13 (72%)**	**7.8 (2.58, 23.58)**	**5.153e‐05**
Scabies	**5 (11%)**	**6.02 (1.12, 32.39)**	**0.020**	1 (6%)	1.12 (0.13, 9.86)	0.921
Eczema (atopic dermatitis)	7 (15%)	1.35 (0.49, 3.70)	0.559	2 (11%)	0.85 (0.18, 4.07)	0.844
Asthma	**9 (20%)**	**3.04 (1.09, 8.53)**	**0.029**	2 (11%)	0.9 (0.19, 4.31)	0.895
Hayfever	4 (9%)	0.97 (0.29, 3.28)	0.959	1 (6%)	0.54 (0.07, 4.40)	0.559
Food allergy	3 (7%)	1.37 (0.31, 6.00)	0.675	1 (6%)	1 (0.12, 8.64)	1
Iron deficiency	9 (20%)	2.23 (0.85, 5.87)	0.099	2 (11%)	0.92 (0.19, 4.44)	0.921
Vitamin D deficiency	7 (15%)	2.02 (0.70, 5.85)	0.189	1 (6%)	0.52 (0.06, 4.27)	0.541
Body mass index	*n* = 45					
Underweight^l^	8 (18%)	1.38 (0.53, 3.63)	0.509	3 (17%)	1.22 (0.32, 4.70)	0.767
At risk of overweight/overweight/obese^l^	24 (53%)	1.26 (0.62, 2.55)	0.518	9 (50%)	1.03 (0.38, 2.77)	0.951
Overweight/obese^l^	22 (49%)	1.65 (0.81, 3.37)	0.165	9 (50%)	1.56 (0.58, 4.20)	0.378
Obese^l^	**15 (33%)**	**2.34 (1.04, 5.2)**	**0.039**	7 (39%)	2.69 (0.93, 7.77)	0.061
Skin examination findings	*n* = 45					
Head lice	8 (18%)	0.93 (0.37, 2.32)	0.882	6 (33%)	2.55 (0.86, 7.55)	0.084
Impetigo (all)	3 (7%)	0.96 (0.24, 3.89)	0.954	3 (17%)	3.46 (0.81, 14.81)	0.079
Scabies	0	0	0.177	0	0	0.449
AD	9 (20%)	1.24 (0.50, 3.03)	0.645	3 (17%)	0.91 (0.24, 3.41)	0.893

*Note*: The bold values are the statistically significant results.

^a^ *Crowded living conditions* defined as > 2.0 persons‐per‐bedroom (PPB).

^b^ *Bedsharing* defined as sometimes or always bedsharing, as compared with never. Bedsharing “by necessity” includes reasons such as poor heating or insufficient beds.

^c^ *Household furry pets* include cats, dogs, rabbits, guinea pigs, and mice.

^d^ *Social housing* refers to government subsidized short and long‐term rental housing; mainly available to people on very low incomes, and who often have experienced homelessness, family violence or have other complex needs. Social housing is made up of two types of housing: (a) public housing, which is owned and managed by State and Territory Governments, and (b) community housing, which is managed (and often owned) by not‐for‐profit organizations. *Transitional accommodation* is a form of social housing offered by community housing providers. Funded by the State Governments. It offers tenancies to people on the housing register with a “very high” or “high and urgent need for housing.” *Temporary accommodation* is a broad term that describes temporary housing for people who are homeless. This includes hostels (i.e., Salvation army), bed and breakfasts, hotels, boarding (with family, friends or other), couch surfing, and so forth [Australian Housing and Urban Research Institute].

^e^ *Frequent bathing* is defined as bathing at least once daily.

^f^ *Use of bathing agent* defined as any bathing agent including soap, shower gel, shampoo, bush medicine soap, soap‐free wash or soap substitute (as compared with just water).

^g^ *Frequent emollient application* defined as at least weekly.

^h^ *Regularly swim* defined as at least monthly.

^i^ *Maternal multi‐parity* defined as ≥ 3 previous pregnancies.

^j^ *Low birth weight* defined as < 2500 g or < 5.5 pounds.

^k^ *Very remote location of birth* defined as remote (MM 6) and very remote communities (MM 7).

^l^ Body mass index (BMI)‐for‐age *Z*‐scores, as per the WHO Child Growth Standards, are used to assess whether a child's growth measurements are in a normal range. Cut‐off points of ≤ 1.0, > 1.0, > 2.0, and > 3.0 are used to indicate underweight, at risk of underweight, overweight, and obese, respectively, in children aged 0–5 years. Cut‐off points of ≤ 1.0, > 1.0, and > 2.0 are used to indicate underweight, overweight, and obese, respectively, in CYP aged 5–19 years.

**TABLE B4:** Atopic dermatitis: Disease associations in urban‐living Aboriginal CYP.

Response category	Ever (past history of) eczema (AD) (*n = 19*)			Current AD (*n = 26*)		
	*n* (%)	OR (95% CI)	*p*	*n* (%)	OR (95% CI)	*p*
Household structure						
Crowded living conditions^a^	1 (5%)	0.41 (0.5, 3.28)	0.386	3 (12%)	1.06 (0.28, 4.03)	0.928
Stable household numbers	18 (95%)	2.43 (0.30, 19.56)	0.391	23 (88%)	0.93 (0.25, 3.54)	0.917
Bedsharing: sometimes or always^b^	12 (63%)	1.03 (0.38–2.81)	0.949	17 (65%)	1.32 (0.53, 3.31)	0.553
Bedsharing by necessity^b^	0	—	—	0	—	—
Household smokers	6 (32%)	0.52 (0.19, 1.47)	0.215	9 (35%)	0.57 (0.23, 1.37)	0.205
Household furry pets^c^	10 (53%)	0.68 (0.26, 1.79)	0.433	14 (54%)	0.72 (0.31, 1.69)	0.451
Social housing, transitional or temporary accommodation^d^	5 (26%)	0.47 (0.15, 1.43)	0.178	7 (27%)	0.44 (0.17, 1.16)	0.094
Social housing^d^	4	0.52 (0.16, 1.76)	0.291	5 (19%)	0.44 (0.15, 1.30)	0.133
Household health hardware						
Absence of a working washing machine at home	0	—	0.3785	0	—	0.303
Plumbing or bathroom maintenance issues at home	2 (11%)	1.00 (0.21, 4.85)	0.996	2 (8%)	0.71 (0.15, 3.36)	0.665
Skin care routine						
Frequent bathing^e^	18 (95%)	0.14 (0.01, 2.41)	0.121	26 (100%)	Infinite	0.509
Use of bathing agent^f^	19 (100%)	Infinite	0.582	26 (100%)	Infinite	0.509
Frequent emollient application^g^	10 (53%)	1.29 (0.49, 3.39)	0.611	**17 (65%)**	**2.61 (1.04, 6.51)**	**0.037**
Regularly swim in a chlorinated pool^h^	1 (5%)	0.41 (0.05, 3.28)	0.386	1 (4%)	0.258 (0.03, 2.03)	0.170
Regularly swim in the ocean^h^	1 (5%)	0.82 (0.10, 6.95)	0.855	1 (4%)	0.56 (0.07, 4.68)	0.589
Use of traditional medicinal plants as part of everyday skin care routine	1 (5%)	0.43 (0.05, 3.46)	0.416	2 (8%)	0.66 (0.14, 3.12)	0.599
Perinatal history						
Maternal age < 20 years at birth	3 (16%)	0.82 (0.222, 3.06)	0.772	5 (19%)	1.04 (0.35, 3.06)	0.945
Maternal smoking in pregnancy	7 (37%)	0.71 (0.25, 1.99)	0.517	9 (35%)	0.55 (0.22, 1.36)	0.193
Maternal multiparity^i^	8 (42%)	1.21 (0.45, 3.22)	0.708	8 (31%)	0.73 (0.29, 1.82)	0.501
Low birth weight^j^	4 (21%)	1.13 (0.34, 3.73)	0.845	4 (15%)	0.72 (0.22, 2.30)	0.578
Very remote location of birth^k^	0	—	—	0	—	—
Past medical history						
Bacterial skin or soft tissue infection (all)	9 (47%)	2.25 (0.84, 5.99)	0.099	10 (38%)	1.58 (0.65, 3.83)	0.309
Tinea (all)	7 (37%)	1.35 (0.49, 3.70)	0.559	9 (35%)	1.24 (0.50, 3.03)	0.645
Scabies	1 (5%)	1.05 (0.12, 9.21)	0.968	2 (8%)	1.78 (0.33, 9.75)	0.501
Eczema (atopic dermatitis)	—	—		**14 (54%)**	**26.13 (8.01, 85.22)**	**1.914e‐11**
Asthma	4 (21%)	2.85 (0.80, 10.13)	0.096	5 (19%)	2.30 (0.73, 7.29)	0.149
Hayfever	**5 (26%)**	**5.53 (1.57, 19.41)**	**0.037**	**7 (27%)**	**6.53 (2.04, 20.04)**	**0.0005**
Food allergy	**4 (21%)**	**8 (1.81, 35.36)**	**0.002**	**4 (15%)**	**5.18 (1.20, 22.29)**	**0.016**
Iron deficiency	3 (16%)	1.23 (0.32, 4.70)	0.762	3 (12%)	0.75 (0.20, 2.78)	0.670
Vitamin D deficiency	4 (21%)	2.42 (0.69, 8.49)	0.158	5 (19%)	2.23 (0.70, 7.09)	0.167
Body mass index						
Underweight^l^	2 (11%)	0.64 (0.13, 3.01)	0.572	2 (8%)	0.43 (0.09, 1.98)	0.269
At risk of overweight/overweight/obese^l^	12 (63%)	1.89 (0.70, 5.12)	0.208	16 (62%)	1.83 (0.77, 4.35)	0.171
Overweight/obese^l^	9 (47%)	1.38 (0.52, 3.63)	0.518	13 (50%)	1.61 (0.60, 3.77)	0.273
Obese^l^	5 (26%)	1.24 (0.41, 3.75)	0.709	5 (19%)	0.80 (0.27, 2.32)	0.678
Skin examination findings						
Head lice	3 (16%)	0.82 (0.22, 3.06)	0.772	3 (12%)	0.52 (0.14, 1.88)	0.315
Impetigo (all)	3 (16%)	3.13 (0.74, 13.36)	0.108	2 (8%)	1.17 (0.23, 5.84)	0.852
Tinea (all)	2 (11%)	0.85 (0.18, 4.07)	0.844	3 (12%)	0.91 (0.24, 3.42)	0.893
Scabies	**2 (11%)**	**14.47 (1.24, 168.27)**	**0.006**	1 (4%)	1.56 (0.16, 15.62)	0.704

*Note*: The bold values are the statistically significant results.

^a^ *Crowded living conditions* defined as > 2.0 persons‐per‐bedroom (PPB).

^b^ *Bedsharing* defined as sometimes or always bedsharing, as compared with never. Bedsharing “by necessity” includes reasons such as poor heating or insufficient beds.

^c^ *Household furry pets* include cats, dogs, rabbits, guinea pigs, and mice.

^d^ *Social housing* refers to government subsidized short and long‐term rental housing; mainly available to people on very low incomes, and who often have experienced homelessness, family violence or have other complex needs. Social housing is made up of two types of housing: (a) public housing, which is owned and managed by State and Territory Governments, and (b) community housing, which is managed (and often owned) by not‐for‐profit organizations. *Transitional accommodation* is a form of social housing offered by community housing providers. Funded by the State Governments. It offers tenancies to people on the housing register with a “very high” or “high and urgent need for housing.” *Temporary accommodation* is a broad term that describes temporary housing for people who are homeless. This includes hostels (i.e., Salvation army), bed and breakfasts, hotels, boarding (with family, friends or other), couch surfing, and so forth [Australian Housing and Urban Research Institute].

^e^ *Frequent bathing* is defined as bathing at least once daily.

^f^ *Use of bathing agent* defined as any bathing agent including soap, shower gel, shampoo, bush medicine soap, soap‐free wash or soap substitute (as compared with just water).

^g^ *Frequent emollient application* defined as at least weekly.

^h^ *Regularly swim* defined as at least monthly.

^i^ *Maternal multi‐parity* defined as ≥ 3 previous pregnancies.

^j^ *Low birth weight* defined as < 2500 g or < 5.5 pounds.

^k^ *Very remote location of birth* defined as remote (MM 6) and very remote communities (MM 7).

^l^ Body mass index (BMI)‐for‐age *Z*‐scores, as per the WHO Child Growth Standards, are used to assess whether a child's growth measurements are in a normal range. Cut‐off points of ≤ 1.0, > 1.0, > 2.0, and > 3.0 are used to indicate underweight, at risk of underweight, overweight, and obese, respectively, in children aged 0–5 years. Cut‐off points of ≤ 1.0, > 1.0, and > 2.0 are used to indicate underweight, overweight, and obese, respectively, in CYP aged 5–19 years.
